# De‐colonizing conservation in a global world

**DOI:** 10.1002/ajp.23258

**Published:** 2021-03-25

**Authors:** Annette Lanjouw

**Affiliations:** ^1^ Great Apes and Gibbon Program Arcus Foundation New York New York USA

## Abstract

Humans form an integral part of most ecosystems on earth. To prevent habitat and species loss and destruction, social justice must, therefore, be at the core of conservation efforts. Traditional conservation education approaches focus on building knowledge, skills, and awareness amongst local communities with the hope of leading to behavior change resulting in the protection of species and ecosystems. The principal drivers of threats to these ecosystems, however, are often not the local people but rather the interests of industry, governments and consumers in distant places. To mitigate and abate the threats to ecosystems, conservation approaches must be both localized and decolonized, including on all the relevant stakeholders. This starts by ensuring that industry, government, and financing institutions have the skills and incentives to avoid harm to the people, wildlife, and ecosystems they exploit, and ensuring that local and indigenous communities are not only informed, but much more engaged in leading the activities that affect them or their land/resources. Essentially, it is the behavior of the global community that must change with respect to the consumption, utilization, and extraction of tropical forest resources and conservation targets must reflect this. Conservation can only be successful when the threats to ecosystems are adequately understood and local people are part of the design and leadership of conservation efforts. This commentary provides specific examples of how conservation education can focus on the drivers of threats, building expertize in the relevant audiences and partners.

## Research Highlights

- Conservation education aims to build knowledge, skills, and awareness of local communities, based on the premise that increasing knowledge and skills would lead to a reduction in threats to conservation targets.
- The main threats to conservation targets are from industrial pressures emanating from distant sources.
- Conservation efforts must, therefore, focus on empowering local communities as well as abating and mitigating threats.

## Graphical abstract

Industrial agriculture (pyrethrum) Rwanda.

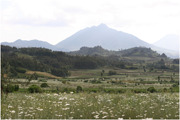

## 1 INTRODUCTION

Conservation of nature can be defined in numerous ways, despite there being overall agreement with the general principles. Some definitions explicitly include humans and their needs whereas others focus only on nonhuman animals, plants and ecosystems. For the purpose of this commentary, we define conservation of nature as preventing the destruction, degradation and decline of species, landscapes and ecosystems to ensure their long‐term survival. Humans are an integral part of these landscapes and ecosystems and are thus included in the focus.

Conservation education is one of the multiple strategies used in wildlife and nature conservation. Although it is interpreted differently by conservation practitioners in different parts of the world, on the whole conservation education is understood to encompass the effort of raising knowledge and awareness to drive behavior change around the utilization, consumption or destruction of species/landscapes/ecosystems as well as the cultivation of support for their conservation and sustainable management. The goal can be the reduction of threats to conservation targets or the building of leadership amongst local communities to empower and enable them to directly manage and deliver on conservation objectives, or to develop viable and desirable careers in conservation. Where unsustainable livelihood practices by local communities are perceived to pose a significant threat to conservation targets, the aim of education efforts is to provide the skills and knowledge required to develop alternative livelihoods that conserve biodiversity and increase human wellbeing and resilience. Conservation education is considered essential to promote conservation policy, create knowledgeable citizens, change people's behaviors, raise funds and recruit new conservation leaders and practitioners (Jacobson & McDuff, 2015).

Historically, conservation education was based on the premise that increasing people's inadequate knowledge and understanding of wildlife/ecosystems would result in them valuing them more, thus encouraging them to support conservation and engage in more sustainable practices. The assumption was that lack of knowledge and understanding was the main factor preventing people from engaging in sustainable practices. Proposed conservation solutions and strategies were often developed from a western perspective and values, without understanding of the beliefs, culture and livelihoods of the local people, and without taking into considerations limits to the opportunities available to them, due to poor infrastructure, poverty, conflict, political or regulatory constraints.

There is growing awareness today that lack of knowledge or understanding is not the main impediment to sustainable practice. Numerous different factors can pose barriers to long‐term and sustainable management of resources and ecosystems, not least of which are the interests of national and multinational companies intent on exploiting natural resources, as well as the governments benefiting from that exploitation. There is increased recognition that local communities have a far greater investment in long‐term and sustainable management of land and resources than external actors who only benefit from extraction. The importance, therefore, of engaging local communities from the very beginning of conservation planning is increasingly valued. This needs to start at the onset of any project development, focus on goals that are meaningful to local people and develop capacity and leadership to implement the conservation programs. Decolonizing the approach to conservation is a necessary step to ensure that conservation programs address conservation threats, are sustainable and focus on both social and environmental justice.

## 2 TRADITIONAL APPROACHES TO CONSERVATION EDUCATION AND THE NEED FOR NEW IDEAS

Conservation education is one tool in a toolbox of different approaches to protect wildlife, biodiversity, and ecosystems. No one approach alone can resolve the complex challenge of ensuring human and nonhuman life can coexist in a sustainable and resilient manner. Conservation education is specifically focused on the knowledge, skills and attitudes required to build support for conservation and effective and sustainable conservation approaches. The threats to wildlife, biodiversity and ecosystems come from multiple sources and at multiple levels, however, and insufficient knowledge or skills is but one challenge that must be addressed.

Human behavior poses the greatest threat to ecosystems and all its wildlife and has led to catastrophic decline in biodiversity and destruction of habitats, pollution, climate change, the emergence of disease and ecological imbalances (IPBES, 2019). Addressing the knowledge gaps to influence human behavior is therefore an obvious strategy towards halting the decline of ecosystem services and loss of biodiversity. It is important, however, that humans and human behavior not be considered too simplistically, as a homogenous category where “education” will lead to changes in behavior. It is important to understand that motivations differ widely depending on location, power and ability to influence the use of land. The focus of most conservation education work, and certainly the case studies described and included here, has been on communities living in close proximity to apes and ape habitat.

Communities in or near forests with apes and ape habitat are critical stakeholders in achieving effective conservation outcomes. Initiatives that engage them, benefit them and address their priorities have a greater chance of long‐term success and conservation impact. It is important to recognize, however, that the people in positions of influence on land‐use, resource exploitation and investment are vital stakeholders to engage as well, and it will require significant investment of effort to build their understanding, skills and incentives for sustainable use, management and conservation of natural resources. These include range‐state governments, industry leaders, governments in end‐user countries and the general public that ultimately demand and consume the resources extracted.

Framing conservation education purely on the communities in or near forests has its limitations and is overly simplistic with respect to addressing the larger conservation challenge. It places the focus and onus on local people and leaders to halt the destruction of wilderness areas and protect wildlife and ecosystems. Biodiversity loss, however, is increasingly related to production and consumption processes situated in other places, whether regional, national or global. The exploitation and extraction of natural resources in distant countries, with little accommodation for the impact this has on local communities and ecosystem health, is a continuation of colonial practices and inequitable power dynamics. Protection of biodiversity cannot only be addressed, therefore, purely through the local protection of sites, with local interventions. Global markets, trade and finance systems drive the threats to tropical ecosystems and the biodiversity in tropical forests. Global value chains shape the choices that local farmers, foresters, fishers or miners make (IDDRI‐Hermès Foundation, 2016) and influence the options they have available to them. Decisions made by governments, together with financing institutions and companies that extract or exploit natural resources (mining, industrial agriculture, logging, paper & pulp, hydro‐electric power and others), whether to allocate land as concessions or remove entire villages to enable land to be converted, subdivided or cleared, can have devastating impacts on the local people and ecosystems affected. Communities are often unable to resist these powerful forces. Although most field researchers and conservationists are all too aware of the threats to wildlife and habitats coming from industrial, commercial and political power‐bases, their focus is often on local communities and issues, due in large part to significant constraints on funding, manpower and mandate. It is vital, however, for the emphasis of conservation education programs to be much broader in scope and focus on a multitude of different audiences.

## 3 IS IT HAVING THE INTENDED IMPACT?

Despite many years of effort, and significant funds invested in conservation education, there have been few systematic studies that have demonstrated its impact on the resilience and survival of species and reduction of threats to wildlife and landscapes. Evaluation effort has primarily gone into assessing the tools, and the proximal changes resulting from the initiatives.

- Did people participate in the initiative?
- Did people understand the information shared?
- Did they remember the information or training that they received?
- Did people utilize the skills, knowledge, training they received?

Few studies have assessed whether or not the activities led to changes in behavior, whether there is cultural transmission of the new behaviors and the behaviors are sustained, and whether the behaviors cumulatively contribute to meaningful reductions in threats to wildlife and landscapes, or whether or not they could be demonstrated to have contributed to resilience and survival of species (Bettinger et al., 2010; Meijaard et al., 2020).

It is therefore challenging to conclude how effective conservation education is as a *conservation* strategy, even if it can be considered an effective *education* strategy. It is admittedly very difficult to draw such conclusions from a single conservation strategy, as the motivators and drivers of hunting, deforestation, trade and other unsustainable utilization of wildlife are rarely simple. And they are rarely purely local. As mentioned above, in most places, the threats to apes and ape habitats come from external sources and pressures that are driven by multiple demands for natural resources and commodities that are sourced in tropical regions of the world. This includes timber and minerals, as well as agricultural commodities like palm oil, coffee, cocoa, and other crops. Focusing conservation education activities on local people living in proximity of forest habitats and wildlife can, therefore, be of value to local communities but may not have any significant impact on threats coming from outside. The main challenge for evaluation will be to link efforts made at the local scale to the international drivers of threats, and linking efforts at global scale (consumer awareness, certification of tropical forest products like the Forest Stewardship Council or the Roundtable on Sustainable Palm Oil) with impact at local scale.

## 4 WHAT ARE WE LEARNING?

### 4.1 The need for an expanded concept of audience for conservation education

Habitat loss and degradation is primarily a result of industrial agriculture, infrastructure development, and extractive industries. Although itinerant (swidden or slash‐and‐burn) agriculture and hunting is a significant threat to forests and wildlife in many parts of Africa and Southeast Asia (Rahman et al., 2017), it is largely influenced by people's lack of formal land tenure and lack of incentives to protect soils and invest in land (Peng et al., 2014). Appropriate land tenure and land rights, including customary tenure, would likely lead to greater levels of investment and security for local farmers in many parts of Africa (Lawry, 2015; Tenaw et al., 2009).

The expansion of industrial agriculture is the primary driver of tropical forest loss (Sodhi et al., 2010). The main crops that are planted in tropical regions that overlap ape distribution include oil palm, peanut, rubber, sugarcane, banana, cacao, coffee, corn, sorghum and tea. In Africa, large‐scale, foreign‐owned plantations were common during the colonial period but decreased over the past 50 years due to insecurity and complex regulatory environments (Smalley, 2013). Over the past 20 years, however, there has been a resurgence of agro‐industrial land investment in Africa, comparable to the investments made in Southeast Asia in previous decades (Lanjouw, 2015). Although local communities are often employed as workers in agricultural plantations, the concessions and permits are awarded by national or regional government officials, and the farming practices and conditions are set by industry. The benefits and profits are also not felt at the local level and workers are generally paid low wages with little job security or protections.

Industrial agriculture as well as extractive industries such as mining, logging and oil/gas exploration are industries that impact large swathes of forest, encroaching on habitats of wildlife but also the land of local communities, who are often displaced or marginalized by the commercial activities. The drivers of expansion of industrial agriculture include increasing global demand for commodities, lower costs and the availability of land considered to be under‐utilized (Arcus Foundation, 2015). The Institute for Economic Affairs estimates that 2.5–3 million km^2^ of land is suitable for food crops in sub‐Saharan Africa, and only 1.8 million km^2^ is currently being cultivated (Boyfield, 2013). Those 2.5–3 million km^2^ of “suitable” land are currently primarily tropical forests, areas of high biodiversity and considered the “lungs of the planet,” as well as the land of indigenous people dependent on the forest and forest resources.

Infrastructure development, from roads, hydroelectric dams, railroads, power‐ and gas‐lines are all expanding across the globe. It is estimated that 90% of the 25 million km of roads that are planned to be built over the next 30 years will be built in developing tropical nations (Dulac, 2013) and cut into tropical forests. Although roads and other infrastructure are frequently built to support economic growth, increasing access to land for agriculture and economic and social integration (Lawrence, 2016; Weinhold & Reis, 2008; Weng et al., 2013), they severely impact ecosystems and species through habitat fragmentation or destruction. Access to new areas of forest, opened through the construction of roads, enables an influx of people to exploit those forests and settle, leading to increases in hunting (Arcus Foundation, in press), agriculture, artisanal mining or logging and other extractive activities. It can also lead to humans coming into contact with novel viruses, bacteria, and diseases that could potentially infect humans, impacting health, economies and wellbeing at the local or even global level. Human settlements in areas adjacent to forests, or in recently cleared areas, can also lead to potential increases in confrontations between wildlife and humans competing for land and resources.

The threats to ecosystems and biodiversity around the world, and particularly in the tropics, is driven largely by economic systems that depend on natural capital to sustain a paradigm of growth. Global trade and increasing wealth around the world have led to increases in consumption, which has led to ever‐increasing levels of extraction from resource‐rich countries. This has often been to the detriment of the people, wildlife and ecosystems in those landscapes. Biodiversity's value has not been recognized or appropriately valued in policy decisions, or the cost of “doing business” (TEEB, 2010). Unsustainable economic activities are the main cause of biodiversity loss and land degradation, but policies insufficiently address the connections between the environment, society and the economy. For human wellbeing to improve as well as ecosystems and biodiversity to thrive, it is essential to address the linkages between these three pillars, rather than looking at them as silos. Effective conservation approaches need to address the nexus instead of focussing solely on policies that promote protected areas or dedicated conservation efforts.

The question, therefore, is who the appropriate audience is for Conservation Education. Although there is no argument that local people and communities who depend on forests and forest products are critical audiences for conservation activities, the gap is in the lack of conservation education focus on other critically important audiences: governments, regional leaders and policy bodies, investors and financial institutions, industry and consumers of tropical forest products. The level of understanding of governments, investors, industry and other audiences is still extremely limited regarding the value of biodiversity, the cost and risk of not protecting it, and the long‐term impacts of the loss of biodiversity and ecosystem degradation. An assessment of investors and asset managers that fund large‐scale infrastructure projects, carried out by Share Action (Share Action, 2019), found that almost none have specific policies regarding biodiversity and there is little systematic engagement on biodiversity related issues. Although investors are conscious of this lack, there exist numerous barriers to their appropriate engagement. These include lack of information on the business case for preserving biodiversity, and the risks/opportunities; inadequate information on species and ecosystems impacts and opportunities for investors; insufficient understanding of how investors could develop an appropriate biodiversity policy and how it can be implemented to have a positive impact. All of these issues could be prime targets for conservation education efforts.

### 4.2 Strategy

#### 4.2.1 Understanding of the drivers of human behavior

As described by others in this journal, the threats to forests and wildlife posed by local people are often motivated by enormously divergent cultures or socioeconomic needs that can vary from place to place (Head, 2017). This is particularly relevant for hunting, an activity that can result from very different motivations. Primates and other wildlife can be a source of food, live animals for trade or as pets, or valued for their parts (bones, fur, skulls) or for different spiritual/cultural traditions. Even the hunting of primates for meat can include multiple motivations, such as bringing food to the family, an economic activity designed to generate cash, required to pay school fees or medical costs, or a cultural activity that is adopted at a certain point in time for ceremonial or traditional reasons. In some places, apes are never hunted due to cultural taboos whereas in other areas they can be considered a prize catch. It is therefore vital to have an accurate understanding of the motivators for different hunters, buyers and traders in different places, and to address conservation messaging and behavior change strategies at the specific issues that are relevant for them.

Many field primatologists and conservationists understand the need to develop clear and nuanced approaches addressing the specific motivators of people. Numerous tools exist to develop such understanding of the specific cultural, economic or temporal contexts that drive certain behaviors. Anthropological and socioeconomic research based on attitude surveys, interviews and questionnaires have been used in numerous sites, enabling people to develop activities that respond to the needs and realities of people on the ground. Social Marketing and Behavior Change strategies are increasingly being used to develop messaging focused on specific behaviors and addressing the drivers of those behaviors.

One of the tools used to build understanding of the impacts of conservation activities and conservation areas on the wellbeing of people living within and around protected areas is the Social Assessment for Protected and Conserved Areas (SAPA) (https://www.iied.org/assessing-social-impacts-protected-conserved-areas-sapa). The underlying assumption is that conserved areas should either contribute to the reduction of poverty or not exacerbate it, and that their benefits and costs should be equitably shared. This principle is contained in the Convention on Biological Diversity and included in the Sustainable Development Goals (Franks & Small, 2016). The SAPA methodology is based on household surveys, using a multistakeholder approach to ensure that key stakeholders are engaged in the design, interpretation of results and development of recommendations for action. In combination with the Social Assessment of Governance and Equity (SAGE) approach described below, conservation programs can build interventions that are co‐owned by the people most affected and respond to their priorities (Roe et al., 2013), thereby highlighting the areas that need to be focused on in conservation education strategies.

#### 4.2.2 Approach and tools

Conservation education was initially framed as a strategy to address what was perceived as a root problem: local communities lacked the knowledge and understanding of the important role of species and ecosystems in safeguarding human livelihoods and perhaps also an understanding of the intrinsic value of different species. It has become increasingly clear that this framing is inadequate. Conservationists and scientists framing and developing conservation strategies have come to recognize that knowledge, attitudes and awareness are often not the main challenge. In many cases, people exploit wildlife/land unsustainably because they do not have alternatives to these activities, even if they know that they are extractive or destructive to nature. Even with all the knowledge and understanding, which often is not lacking, they are unable or unwilling to change their behavior due to other factors.

The emphasis of conservation efforts has evolved to a more nuanced understanding of the issues, and the critical relationship between social, economic and environmental issues. The focus has turned to creating alternative and sustainable livelihood activities that generate benefits to humans, coupled with halting the unsustainable destruction and consumptive exploitation of wildlife and natural resources. The conservation education component of these efforts is focused on developing the skills, tools and willingness to adopt these more sustainable activities and ensure that the most vulnerable people have access (pro‐poor development) and are included. A major emphasis has been on creating value for wildlife and natural resources, so that people have a stake in their continued existence and there is motivation for protecting and managing them sustainably. The most well‐known of these strategies is the development of wildlife tourism, based on the premise that healthy and viable populations of wildlife are more valuable for generating income than consumptive utilization. In some parts of the world, sustainable consumptive utilization was an additional strategy, such as trophy hunting or capture for sale. Such an approach can be characterized as “if it pays it stays.” Wildlife or landscapes must be deemed economically valuable to be conserved. Yet in many parts of the world, this is not possible. Although the “pay to stay” approach has dominated the conservation paradigm in much of Southern and Eastern Africa, it has not necessarily been relevant or appropriate in other parts of the world, and even considered to be in direct conflict with other approaches. Proposals to enable consumptive utilization of elephant or rhinoceros for their ivory and horn, for example, has been seen as a significant threat to conservation efforts of elephant and rhinoceros in other parts of Africa (Bauer et al., 2020).

Careful situation analyses that identify the various pressures exerted on wildlife and ecosystems and highlight the proximal and distal drivers of those influences, are necessary to formulate effective conservation strategies. Different tools exist to help people determine who the relevant actors are, and which conservation measures are most appropriate for a site. Examples of such tools include the Site‐level assessment of governance and equity (SAGE) for protected areas and conservation activities. This method, led by the International Institute for Environment and Development (https://www.iied.org/site-level-assessment-governance-equity-sage) and implemented in collaboration with numerous conservation organizations, assesses governance and equity issues in conservation landscapes and the associated activities, identifies challenges to support planning and provides information on monitoring and management oversight. It is based on the good governance and equity principles related to equity in recognition, procedure, distribution and conservation impact (Franks et al., 2018).

An important principle to take into consideration and use to guide conservation actions that involve different human communities, recognized in the United Nations Declaration on the Rights of Indigenous Peoples is the right to Free, Prior and Informed Consent (FPIC). It allows local and indigenous communities to give or withhold consent to a project that may affect them or their land/resources, based on information provided freely and given without coercion, manipulation or intimidation; provided sufficiently in advance of commencement of activities, and including all the appropriate and relevant information. FPIC enables communities to negotiate with power and rights the conditions under which a project will be designed, implemented, monitored and evaluated.

These principles must guide the manner in which communities engage in conservation activities that discourage, prevent or prosecute people for utilizing resources that they depend on for their survival or wellbeing, yet in many cases they are not adequately integrated into conservation programs. Conservation education, despite the best of intentions, does not always focus on the rights, governance and equity issues that local and indigenous peoples need to be full and respected partners. The lack of adequate incorporation of these approaches is often a cause of failure or disgruntlement. In addition, conservation practitioners are not always fully informed or educated in these approaches and despite genuine good intentions, programs frequently do not fulfill their potential due to challenges in the relationships with local people, or faults in the design of conservation interventions, including conservation education.

### 4.3 Evaluation

Due to the diverse interpretations and objectives of conservation education, and without understanding the barriers to behaviors conducive to conservation outcomes, it is very challenging to measure effectiveness and impact. Much of the understanding and interpretation of the value of conservation education efforts has been anecdotal and based on assumptions and expectations. The assumption that insufficient knowledge and understanding form the main challenges, is often misplaced.

Rigorous evaluation of the impact of conservation education activities is relatively rare, at the local as well as global level, although some notable exceptions exist. Some of the papers included in this journal demonstrate rigorous approaches to evaluation (e.g., Bettinger et al.). As stated earlier, however, the focus is more frequently on the immediate changes, rather than the longer‐term impacts on behavior and the threats to wildlife (Baylis et al., 2015, and Conservation Measures Partnership https://conservationstandards.org/about). This lack of evaluation is in part a result of the difficulty of attributing change to any one intervention when the drivers of threats and behaviors are complex and different between people, between sites, and over time. Effective conservation requires numerous strategies and approaches addressing the needs at local, political, institutional and global level. It is often very difficult to attribute any measurable change to any one particular intervention. At the same time, evaluation costs time and money and is often not the priority for underfunded and under‐resourced organizations. It is also true that the donor community who provide a significant portion of the institutional funding for conservation activities do not enable objective evaluation and assessment of failure. The funding environment is highly competitive, and resources are far from adequate to fund all the work and the different approaches required. Conservation organizations often feel obligated to present their work as highly successful to convince donors to invest in that work. This does not incentivize a critical analysis of interventions, failures and learning from experience and instead drives organizations to present countless anecdotes of success. From the perspective of the donor, this leads to confusion and at times an erosion of trust, as it is clear that despite investment of funds and collection of success stories, biodiversity is facing cataclysmic declines and loss of forest cover is accelerating world‐wide.

Although challenging, it is important to the entire conservation sector to have a more honest and rational approach to evaluation of conservation interventions, including components focused on education. It is vital to link local, regional and global efforts in conservation and to demonstrate the differential successes of alternative approaches and strategies. Despite the availability of various tools to measure success at the local level, there are still very few approaches that link direct threats with the drivers of threats and the effectiveness of strategies to combat them.

## 5 CONCLUSIONS

Conservation education is one of many approaches deployed to support the long‐term protection of wildlife, ecosystems and landscapes. It has an important role to play in combination with numerous other tools and strategies. Assessing and evaluating the effectiveness of conservation education has had limited success, although efforts are increasingly being focused on measuring impact to build greater levels of understanding. Conservation education is premised on the assumption that improving knowledge, skills and attitudes is vital to changing behavior and reducing threats to wildlife and ecosystems. Although it is unquestionable that it is an important component of any strategy, it cannot be considered adequate and sufficient for confronting the decline of biodiversity and damage to ecosystems. Lack of alternatives and choices are often the main reason why people engage in practices that are destructive of nature. Numerous drivers, which can be economic, political, cultural or religious, will influence people's choices and behaviors, and it is critical to have nuanced understanding of the way in which the drivers affect people's choices and lifestyles. Effective conservation education strategies need to take these factors into consideration.

Conservation education has been largely focused on local communities living in or adjacent to the areas being protected. The reality on our globalized world, however, is that these communities often have far less influence over conservation success than other actors far removed. The gaps in knowledge, understanding and skills in the sectors that have disproportionate influence on wildlife and ecosystems are enormous. Governments, the corporate and finance/investment sectors are often ignorant of the presence, value and vulnerability of wildlife in the areas that they target for development or investment and have very little understanding of the opportunities and strategies for their protection. They are frequently unaware of the realities for local people who depend on these ecosystems for their survival. Conservation education needs to carefully consider how it can influence behavior of people that are geographically far removed but enormously influential over the forests and apes it aims to protect.

## AUTHOR CONTRIBUTIONS

**Annette Lanjouw**: conceptualization (lead); writing original draft (lead); writing review & editing (lead).

### PEER REVIEW

The peer review history for this article is available at https://publons.com/publon/10.1002/ajp.23258.
